# Total Endovascular Aortic Arch Repair with Branched Graft

**DOI:** 10.1055/s-0039-1694014

**Published:** 2019-11-26

**Authors:** Ilaria Franzese, Giuseppe Petrilli, Giovanni Puppini, Daniela Bacich, Vincenzo Giambruno, Giuseppe Faggian

**Affiliations:** 1 Division of Cardiac Surgery, University of Verona, Verona, Italy; 2 Department of Radiology, University of Verona, Verona, Italy; 3 Cardiology Unit, Madonna della Salute Hospital, Porto Viro, Italy

**Keywords:** endovascular aortic arch repair, branched TEVAR, multibranched graft

## Abstract

In selected cases, the utilization of branched endografts for the treatment of aortic arch aneurysms could be a safe and advantageous alternative to high-risk procedures such as open total aortic arch replacement or hybrid arch repair. We present the case of a 70-year-old man with saccular aneurysm of a bovine aortic arch which was endovascularly treated using a double-branched custom-made aortic endoprosthesis based on the Relay NBS (Non-Bare Stent) Plus platform intended for zone 0 deployment. The postoperative clinical course was uneventful. The postoperative computed tomography scan showed a good result of the implant. The patient was discharged 6 days after the procedure.

## Introduction

The endovascular repair techniques developed for aortic arch disease offer an alternative treatment to open surgery, especially for patients with a high-risk surgical profile. Hybrid aortic arch repairs have been developed combining the use of thoracic endovascular aortic repair (TEVAR) with a conventional elephant trunk repair or with an open surgical debranching of the cerebral vessels. 1 2

To further minimize the perioperative risks and the potential negative impact of these complex procedures on long-term outcomes of patients with aortic arch aneurysms, a concept of total endovascular repair for aortic arch disease has recently emerged. In fact, branched aortic endografts have been developed for this purpose and are currently undergoing clinical investigation.

We present the case of a patient with a large saccular aneurysm of the aortic arch successfully treated with a custom-made double-branched endograft.

## Case Presentation

**Fig. 1:**
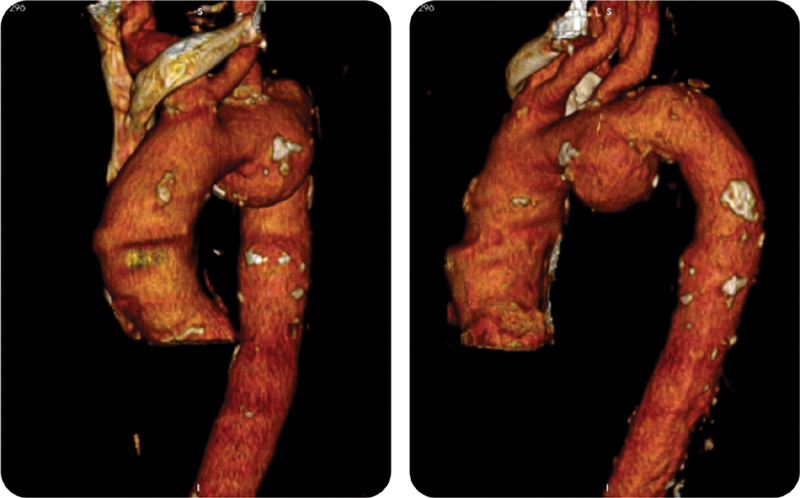
Preoperative computed tomography assessment scan.

A 70-year-old man with a history of smoking, hypertension, previous thyroidectomy, and kidney cancer with a single lung metastasis was referred to our institution for a voluminous saccular aneurysm of the aortic arch of 4.8 cm in diameter which was incidentally found during a computed tomography (CT) of the chest performed because of pneumonia. The aneurysm was located just opposite to the origin of the left subclavian artery and it contained a thrombotic plaque and demonstrated thin calcifications of the aortic wall ( Fig. 1 ).

The patient had an American Society of Anesthesiologists score of 3 and a Society for Vascular Surgery/American Association for Vascular Surgery medical comorbidity score of 5. After a multidisciplinary evaluation, the patient was deemed eligible for branched TEVAR of aortic arch.

**Fig. 2:**
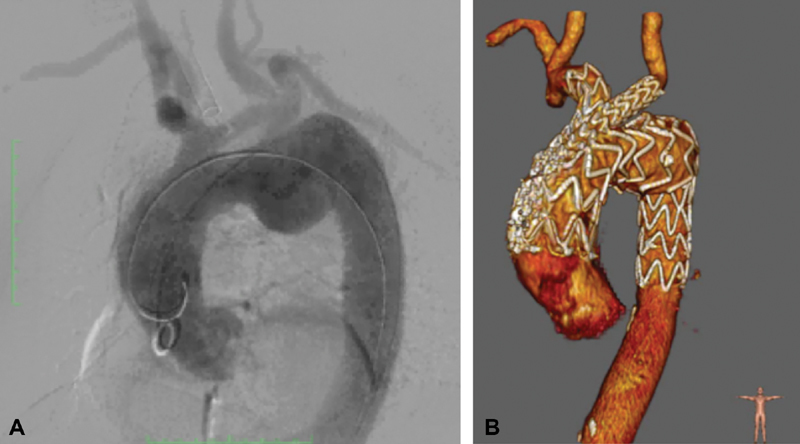
Angiography (panel B) and computed tomography scan—three-dimensional view (panel A) pictures following the procedure.

**Fig. 3:**
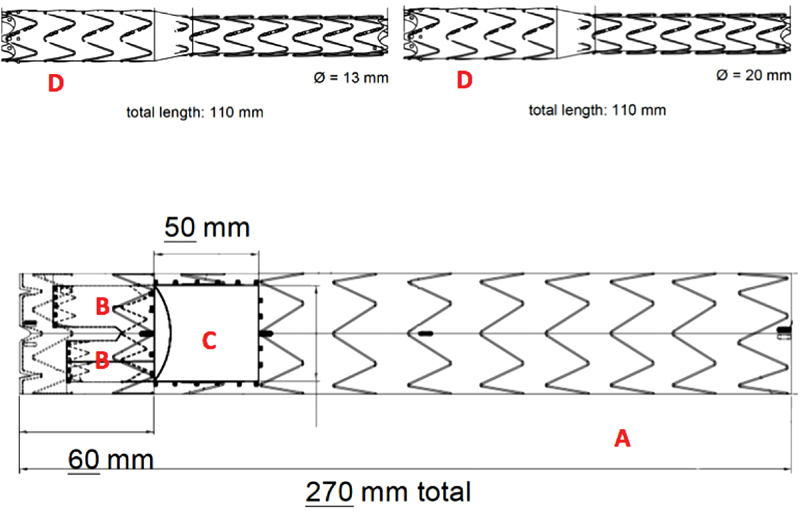
Graft components: main graft ( **A** ), inner branches ( **B** ) and fenestration ( **C** ) for graft branches ( **D** ).

Analysis of the preoperative CT scan showed that the saccular aneurysm protruded from the anterolateral aspect of the aortic arch opposite the origin of the left subclavian artery. The proximal seal zone in ascending aorta was 40 ± 2 mm, the aneurysmal length was 70 mm and distal seal zone in descending aorta was 31 mm and >25 mm in length ( Fig. 2 ). The CT also demonstrated a short common bovine trunk, which required two branches for innominate artery and left common carotid artery (CCA) ( Fig. 3 ).

The ascending aorta and the femoral and iliac arteries were normally sized.

The procedure was performed in our angiographic interventional suite.

Both common carotids and the right femoral artery were surgically exposed through bilateral cervical incisions and right inguinal incision, respectively. The left femoral artery was utilized for percutaneous access. A transvenous pacing lead was easily introduced through the common femoral vein to ensure cardiac output reduction during graft deployment.

A customized Bolton Relay NBS (Non-Bare Stent) Plus (Bolton Medical Inc. Sunrise, FL and Barcelona, Spain) endovascular prosthesis (dimension 46–36 × 270 mm, according to preoperative CT scan data) was inserted over a curly stiff wire, with floppy tip sited in the apex of the left ventricle.

The graft was deployed, during rapid cardiac pacing, with proximal landing zone in ascending aorta (Zone 0) and distal end in thoracic descending aorta. Because the CT demonstrated a competent circle of Willis, the left subclavian artery was intentionally covered by the main stent graft allowing a straightforward and complete endovascular procedure with the utilization of a double-branched prosthesis.

The main graft, based on Relay NBS Platform, had a fenestration located at 60 mm from the proximal end with two antegrade internal branches, sized 12 and 12 mm.

The precurved inner catheter of the delivery system allowed the device to self-align with the anatomy, placing the big fenestration oriented toward the top of the arch. The fenestration was identified by a series of dot-shaped markers and by a dumbbell marker for longitudinal alignment.

The internal branches had anchors for a locking mechanism to prevent branch migration. The branches were cannulated in a retrograde fashion via the carotid arteries.

The first inner branch, for the innominate artery itself, was cannulated from the right CCA and the wire was positioned in the left ventricle. The distal CCA was clamped to avoid air or debris embolization, and the first branch (20 × 110 mm) was deployed. A control angiogram was performed before removing the sheath and closing the arteriotomy. The distal inner branch was cannulated from the left CCA and the second branch graft (13 × 110 mm) was deployed in a similar manner.

Angiography confirmed complete exclusion of the aortic arch aneurysm ( Fig. 2 ), with no endoleaks and normal patency of the epiaortic vessels, while the covered left subclavian artery was perfused retrogradely from the ipsilateral vertebral artery.

Time of procedure was ∼3 hours. No early neurological injury occurred, and the patient was extubated within 3 hours. His intensive care unit stay length was 1 day.

The patient had an uneventful postoperative clinical course and was discharged 6 days after the procedure.

Three- and six-month CT follow-up showed complete exclusion of the aneurysm, patency of the stent graft, and no endoleaks.

At 1-year clinical follow-up, the patient was well and no late vascular or neurological complications occurred.

## Discussion

Saccular arch aneurysms are uncommon and have a higher risk of rupture than fusiform aneurysms. 3

Conventional open surgery has been the mainstay of treatment options for aortic arch aneurysms, but it requires cardiopulmonary bypass and deep hypothermic circulatory arrest, and it is associated with significant morbidity and mortality. 4

The development of TEVAR can simplify the treatment of both fusiform and saccular aortic aneurysms. In fact, endovascular treatment of such lesions has been shown to reduce operative time and length of hospital stay and improve perioperative morbidity and mortality. 5

In 2009, Cherrie Abraham implanted the first multibranched graft for a total arch repair. 6

In 2016, the analysis of Spear et al reported 100% technical success and 7.4% major stroke in 27 patients who underwent aortic arch repair with inner branched endografts. 7

The first experience with the Bolton Relay Plus arch branch device was presented in 2015, with 100% operative success in 26 patients. 8

In our case, we used a modular stent graft, which was designed with two branched stent grafts, one for the innominate artery and the another one for the left common carotid artery.

Branch tunnel position on the desired greater curve is permitted with Bolton's patented self-orienting precurved nitinol guidewire lumen, which enables alignment of the arch graft's cannulation window to the primary curve of the arch. 8

In our opinion, this technology allows a wider degree of manoeuvrability during the deployment of the device, and in particular during the alignment of the fenestrations and the epiaortic vessels.

However, one of the major drawbacks of the devices currently available for endovascular treatment of aortic arch aneurysms remains the protracted time required to prepare the custom-made graft, which may not be available for urgent intervention. Although promising, this technology for endovascular treatment of the aortic arch is not supported by generous data. Randomized clinical trials to compare this approach to aortic arch disease with conventional and hybrid strategies are necessary.
